# Development and Validation of Questionnaire to Assess Knowledge about Cervical Cancer among Women Aged 20 to 65 years in Oman

**DOI:** 10.31557/APJCP.2021.22.1.69

**Published:** 2021-01

**Authors:** Aisha Al Saad

**Affiliations:** *Department of Studies and Research, Directorate General of Planning and Studies, Ministry of Health, Muscat, Oman. *

**Keywords:** Knowledge- cervical cancer- cancer prevention- women- questionnaire validation

## Abstract

Objective:

This study aimed to develop and validate a questionnaire to assess awareness of cervical cancer, its risk factors, and methods of prevention among Arabic-speaking women aged 20 years and over. **Methods:** The study took place at primary healthcare institutions, Al Buraimi Governorate, Oman, between November 2018 to February 2019. In phase I, seventy items concerning cervical cancer and its prevention were generated through a literature review. In phase 2, the questionnaire was validated through calculating the content validity index (CVI) for both item level (I-CVI) and the scale level (S-CVI), in this phase a shortened English questionnaire of 55 items was formed, then rigorously translated to the Arabic language in phase III. The questionnaire was tested for reliability in two stages: A pilot and a large field test in phase IV. **Results:** A total of 55 out of 70 items formed the final version of the questionnaire. The final instrument had an S-CVI/Ave of 0.92. The questionnaire called the Knowledge in Cervical Cancer and Prevention Methods 55-items (KCCPM-55). The Cronbach alpha coefficient was 0.940 for the whole questionnaire, and ranged between 0.57 to 0.93 for each of the domains. Test-retest reliability was examined in a subsample of the total participants sample (r = 0.769, p < 0.001). **Conclusion: **The KCCPM-55 has been successfully developed in the Arabic language and found to be a valid and reliable instrument for assessing the level of knowledge about cervical cancer and prevention methods among women aged 20 to 65 years in Oman.

## Introduction

Cervical cancer is a major public health problem for adult women. The incidence varies greatly worldwide. It continues to represent the fourth most common malignancy affecting women all over the world, and the second most common cancer in developing areas with an estimated 570,000 new cases and 311,000 deaths from the disease occurred in 2018; more than 80% of the new cases and deaths occurring in low and middle-income countries (Arbyn et al., 2019; World Health Organization, 2020). The predictions in the Middle East, indicate that this region will experience an increase of mortality incidence which will approximately reach 181% over the next 15 years (Dey and Soliman, 2010), which means that the global burden occurs in developing areas.

The incidence of cancer is rapidly increasing in Oman (Al-Lawati et al., 2008). Current estimates of incidence and mortality indicate that every year 77 women are diagnosed with cervical cancer and 41 die from the disease. Cervical cancer ranks as the 3^rd^ most frequent cancer among women aged 15 to 44 years in Oman with a crude incidence rate of 4.7 and a mortality rate of 2.5 respectively ( Bruni et al.,2019).

Cervical cancer is mostly caused by the infection of Human Papilloma Virus (HPV) (Tezcan et al., 2014). This virus has been implicated in 90% of cases of cervical squamous cell cancer (Daniyal et al., 2015). HPV 16 and HPV 18 types of the virus cause approximately 70% of all cervical cancer cases (Tewari and Monk, 2012). Recent study found the prevalence of Human Papilloma Virus in Oman is 17.8% (Al-Lawati et al., 2020).

Cervical cancer is fatal if untreated but preventable with the aid of primary and secondary prevention methods. In countries where the resources exist, many primary and secondary preventative approaches have been optimized to ensure cervical cancer prevention. Secondary prevention is based on cervical screening. The Papanicolaou (Pap) test is the most widely used cervical cancer screening test. It used for screening 80% of women with cervical cancer. The primary prevention is based on prophylactic vaccination against the major cause of cervical cancer, the carcinogenic human papillomavirus types 16 and 18. However, the burden of cervical cancer is very high (Daniyal et al., 2015), because the governmental effort to inform the population about cervical cancer is still lacking. A very poor level of knowledge about cervical cancer is evident among general populations, as well as health professionals (Giles and Garland, 2006; Ortashi et al., 2013). Implementing both methods of prevention can reduce the death rate from cervical cancer, however, it cannot be attained without improving community awareness of this disease which improve early recognition and prompt treatment (Sankaranarayanan and Ferlay, 2005).

In Oman, no screening program yet instituted at primary health care which invites high-risk women for regular cytological screening. The HPV vaccine is also not yet introduced in the national immunization program. Prophylaxis and early detection services play a vital role. However, the uptake of those services will remain low with a lack of awareness about cervical cancer. The assessment of knowledge with a valid and reliable instrument is the first step in creating interventions to raise knowledge and increase the uptake of cervical cancer screening services. Therefore, this study aims to develop and validate a questionnaire used to assess women’s knowledge about cervical cancer, its risk factors, and prevention methods. Assessing the level of knowledge at a younger age is imperative because of the relatively lower marriage and childbirth ages in Oman (Islam et al., 2013). The availability of an Arabic questionnaire that widely assesses the knowledge related to cervical cancer might help to improve the way we fight this preventable malignancy by having a more comprehensive overview of the level of knowledge among the women population.

## Materials and Methods

The method followed used the basic principles for questionnaire development suggested by the European Organization for Research and Treatment of Cancer (EORTC) Quality of Life Group guidelines (Johnson et al., 2011). This method has been successfully applied by Jaglarz et al., (2014) to create the Cervical-Cancer-Knowledge-Prevention-64 questionnaire used for assessing the level of knowledge about cervical cancer among school girls and female students in Poland. The current study followed the same process for questionnaire development, however, the questionnaire is initially constructed in the English language based on the reviewed literature and then translated and tested in the Arabic language. 

The questionnaire was developed in four phases: 1) item generation; 2) construction and validity of the questionnaire; 3) translation of the questionnaire; 4) field testing. A panel of content experts were requested to assessed the content validity of questionnaire in a two-round evaluation before conducting the field test. The study was approved by a Research Ethical Review and Approval Committee (RERAC) at the Ministry of Health. The questionnaire was accompanied by a consent form and a cover letter explaining the purpose of the study and respondents’ anonymity and voluntariness.

*Phase 1: Item generation*

A literature search for studies discussing the issues concerning cervical cancer and its prevention was conducted before designing the questionnaire. After a thorough literature review, the following four main domains of knowledge were generated: 1) general knowledge about cervical cancer; 2) risk factors associated with the disease; 3) knowledge about primary prevention; 4) Knowledge about secondary prevention. Many validated tools that cover a wider spectrum of knowledge assessment relating to cervical cancer were also reviewed (Donders et al.,2007; Jaglarz et al., 2014; Onan et al.,2009). The first version of the questionnaire included 10-15 items about each domain, depending on how relevant the items seemed. A list of 70 items was generated from the literature review.

*Phase 2: Construction and Validity of the questionnaire*

The scale was developed with 70 items. A panel of 8 content experts (four academicians and four practitioners) experience in the area of gynecology were asked to evaluate the scale in two rounds. Their expert judgment on the relevancy of each item to the corresponding domain was requested in the first round , using a 4-point content validity index (CVI) (1, not relevant, 2, Somewhat relevant, 3, quite relevant 4, highly relevant) (Davis, 1992). All domains of questionnaire were defined ( Table 1). The experts were requested to critically review each domain and provide score on each item independently. Their assessment of translation and clarity of items were also requested in the second round. This has resulted in refining the questionnaire domains and its items. 

Content validity index was calculated both for item level (I-CVI) and the scale level (S-CVI) .Content validity index at item level (I-CVI) was calculated by totaling the number of experts giving a rating of 3 or 4 for each item divided by the total number of experts ( Polit et al. 2007). The CVI cut-off point was set at 0-78 (Lynn 1986). The items that had CVI ≥ 0.78 were considered as relevant, and items below 0.78 were eliminated. Based on item relevance the number of items decreased from 70 to 55. Average approach was used to compute the content validity of the overall scale S-CVI/Ave . The index of average congruity was judged according to the standard which is advised by Waltz et al., (2005). 

The final tool consists of 55 items (4 concerning demographic data), and the remaining 51 items were divided into four main domains, namely general knowledge about cervical cancer ( 6 items), knowledge about risk factors associated with the disease (17 items), knowledge about primary prevention with subdomains: 1) lifestyle (8 items); HPV vaccine (7 items) and knowledge about secondary prevention with subdomains: 1) symptoms of cervical cancer ( 9 items); 2) cytological screening (3 items). 

*Phase 3: Translation of the questionnaire*

**Figure 1:**
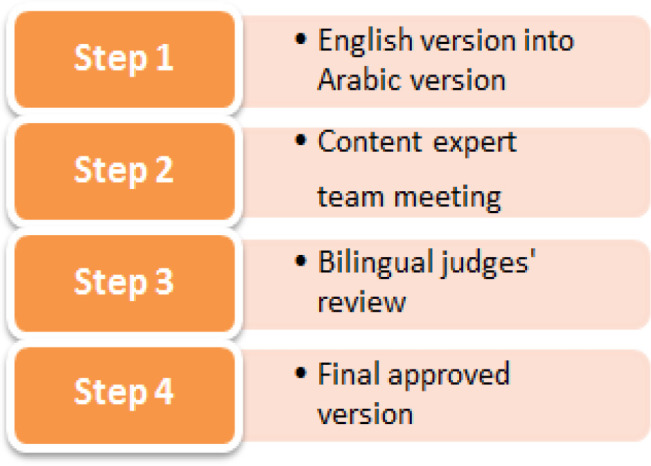
The Translation Process of KCCPM-55

The current study followed similar translation and adaptation process implemented by Alanazi et al., (2017) for adapting the hospital consumer assessment of healthcare providers and system (HCAHPS) questionnaire in the Arabic context (Figure 1). The translation was accomplished by following the four-step translation framework: (1) translation of English version into Arabic by a qualified bilingual translator: (2) The Arabic version was translated back to English and then reviewed by the panel of content experts in the second round to ensure that the translation did not change the meaning of the content (content equivalence): (3) both the Arabic and English versions were given to a qualified bilingual translator for linguistic validation and identify the similarities and differences between the two versions (semantic equivalence). In this step, the discrepancies between the two versions were assessed, to ensure both versions are identical in content and meaning (conceptual equivalence) using the bilingual translation method. Some questions touched an important subject of women’s sexuality; therefore, the appropriateness of wording and potential misinterpretation was considered to achieve the final item wording of the questionnaire (cultural validation). 

*Phase 4: Field Testing of the questionnaire*

Psychometric properties were initially assessed by a pilot study in June 2018. A sample of 70 Omani women attending Al Buraimi hospital were randomly and voluntarily enrolled in the study. The researcher was available to explain to participants the types of questions included in the questionnaire and to ensure that participants understood all sections of the questionnaire, particularly the section which ranks the relation between the identified risk factors and the occurrence of cervical cancer. Respondents were asked to rank the relation on a 6-item Likert scale [never=0, very little=1, little=2, somewhat=3, much=4, and great=5]. The researcher assessed the participants’ understanding of the answers they were choosing. The researcher also asked the participants about any words, phrases, or questions they did not understand, or found unacceptable. All their comments were taken into consideration when forming the final version of the scale. Data were analyzed for internal consistency.

A large field survey was carried out between November 2018 to February 2019 in eight primary healthcare centers in Al Buraimi governorate by using the newly validated Arabic questionnaire, called KCCPM-55. A proportional sampling was carried out to collect data from approximately 5% of Omani women aged 20 to 65 years in Al Buraimi governorate. The sample proportionate to the number of women aged (20-65 years old) living in the catchment area of the eight primary healthcare institutions available in Al Buraimi governorate. The total sample was 1000 participants, assuming a 95% confidence level and a margin of error 3%. A website size calculator was used “Raosoft” to calculate the study size, then a proportional size approach was implemented to calculate the size from each healthcare institution. A woman attending the healthcare center for any reason at the time of data collection were approached to participate in the study if they meet the inclusion criteria: Omani women who aged 20 years and over, women who can read and write, to understand the consent form and fill the questionnaire, women who consent to participate in the study, and women who do not have cervical cancer, thus, women who are diagnosed with cervical cancer and/or receiving cancer treatment were excluded from the study.

The self-administered questionnaires were distributed to the participants through the nurse in-charge in each primary healthcare centers. The completed questionnaires and signed consent forms were inserted in two separate boxes placed at the head of the department office. Data were coded and entered into a computer using two of the latest versions of prepared packages of statistical analysis namely Statistical Package for Social Sciences (SPSS) version 25.0 for Windows and Minitab version 18.1. All these programs used in different stages of data processing to process the raw data obtained from the questionnaires.

The researcher used descriptive statistics to describe the participants’ characteristics, scores of cervical cancer’s general knowledge, risk factors, knowledge of primary and secondary prevention. Internal consistency reliability was assessed by computing Cronbach’s alpha and Kuder -Richardson 20. Test-retest reliability was examined in a subsample of the total participant sample by computing the Pearson correlation coefficients. 

## Results

The Scale-level content validity score was calculated by using averaging approach (Polit and Beck, 2006). 

The S-CVI/Ave was calculated by first multiplying the number of experts by the number of items which returns the total number of possible item-by expert ratings is 560 ( 8 experts X 70items); 517 of them indicated relevance (I-CVI ≥ 0.78). So the S-CVI/Ave was 517/560=0.92. 

The I-CVI for all the items ranged from 0.63 to 1. Based on item relevance, a total of 15 items were considered as irrelevant (I-CVI < 0.78). The number of items decreased from 70 to 55. The final version then translated to the Arabic language and then back into English. The Arabic version was finally approved by the panel of content experts in the second round. After the content validation, the Arabic version was tested for reliability in two stages: the pilot and large field testing. The results of Cronbach’s Alpha tests showed that the alpha coefficient of the KCCPM-55 was 0.935 in the pilot study with a range between 0.54 to 0.80 across the four domains of the scale, as presented in Table 2. Likewise, the alpha coefficient of the questionnaire in a larger sample showed almost the same results except the risk factor domain showed remarkable improvement in internal consistency, it raised from 0.70 to 0.93. The Cronbach’s Alpha across the other three domains were as follows: 0.57 for general knowledge about the disease; 0.73 for knowledge about primary prevention; 0.70 for secondary prevention (Table 2). A sample of 70 participants used to evaluate test-retest reliability. All completed the questionnaire again 2 weeks after their initial completion. Pearson coefficient for simple linear correlation found a significant relationship between the two cervical cancer knowledge scores (r = 0.769, p < 0.001).

A total of 791 women (79.1%) were recruited to take part in the large field study. Slightly less than a half of the respondents (48%, n= 380) were between 31 and 40 years at the time of the survey, followed by 310 (38.2%) were between 20 and 30 years old, and 93 (11.8%) of them were between 41 and 50 years. Only 1% (n=8) of the women who participated in the survey were above 50 years of age. Concerning work status, the majority of the respondents were not working (n=442, 61.3%). More details on the demographic characteristics can be found in Table 3. 

**Table 1:** Definitions of Proposed Domains of Cervical Cancer Knowledge

Domains	Definition
General Knowledge about cervical cancer	Knowledge about prevalence of disease, fatality, the causal role of infections in cervical cancer, preventability of disease.
Risk factors associated with the disease	Knowledge about the risk factors of cervical cancer.
Primary prevention	Knowledge about two domains:
	1-Life style factors that reduce the risk of cervical cancer.
	2-Knowledge about Human Papillomavirus (HPV) vaccination.
Secondary prevention	Knowledge about signs and symptoms of cervical cancer and knowledge about screening based on the Pap test.

**Table 2:** Reliability Measures and Coefficients for 4 Sub-Scales of a Pilot (n=70) and Large Field Study (n=791)

Domains	Measure	A pilot study (n-70)	Large field Study (n=791)
		Coefficient	Coefficient
General Knowledge about cervical cancer	Cronbach’s Alpha	0.54	0.57
Knowledge about Risk factor and the occurrence of the disease	Cronbach’s Alpha	0.70	0.93
Knowledge about primary prevention	Cronbach’s Alpha	0.80	0.73
Knowledge about secondary prevention	Kuder-Richardson 20	0.74	0.70
Overall value		0.935	0.94

**Table 3:** Sociodemographic Characteristics of Participants (N=791)

Characteristics	n (%)
Age	
20-30	310 (39.2)
31-40	380 (48.0)
41-50	93 (11.8)
Above 50	8 (1)
Educational Level	
No education	5 (0.6)
Primary	37 (4.7)
Secondary	327 (41.3)
Tertiary	422 (53.4)
Marital Status	
Single	100 (12.7)
Married	663 (83.8)
Other (divorced or widow)	28 (3.5)
Work status	
Working	306 (38.7)
Not working	485 (61.3)

## Discussion

The content of the KCCPM-55 is the result of extensive literature review and experts’ degree of agreement regarding the content of the proposed instrument. The scale was initially developed with 70 items and based on item relevance, the number of items has decreased. A total of 55 items had CVI ≥ 0.78 which, based on Lynn ‘s advice (1986), was considered as a relevant items. The final instrument had an S-CVI of 0.92. It is advised to determine a scale as having excellent content validity, it would contains a minimum I-CVI of 0.78 (Lynn 1986) and S-CVI/Ave of 0.90 or higher ( Waltz et al. 2005). The KCCPM-55 has been successfully translated to the Arabic context by following a model of a back-translation process. This systematic procedure was needed to ensure the quality of translation outcomes. Results from the pilot study show that it is feasible to administer the Arabic version of the KCCPM-55 questionnaire to a group of Omani women aged 20 years and over. All respondents found the questions acceptable and clear.

This study touched an important subject of women’s sexuality, however, the respondents did not reject taking part in it, as the questionnaire is a self-administered tool. Many self-administered and interview-based questionnaires were used in the Arabic speaking population to assess knowledge about cervical cancer (Al-Meer et al., 2011; Alsaad et al., 2012; Jassim et al., 2018; Jradi andBawazi, 2019). All these studies focused on attitudes of women towards the Pap test. In contrast, KCCPM-55 questionnaire is developed to assess a wider spectrum of knowledge relating to cervical cancer. The tool has focused not only on women knowledge about cytology screening, but also it evaluates women’s’ knowledge about risk factors associated with the disease, lifestyle factors affect a woman’s risk of cervical cancer, knowledge about the HPV vaccine, and knowledge about symptoms of cervical cancer. Using this approach provides a broad overview of the level of knowledge about cervical cancer, which will contribute to supporting efforts to promote the health of women in Oman. 

A cross-sectional study with a sample of 791 women has proven the efficacy of the KCCPM-55 questionnaire. The study results indicated that the KCCPM-55 showed high overall reliability (Cronbach’s Alpha 0.940) and a high degree of consistency to that proposed by Hinton et al., ( 2014), in which the minimum internal consistency value as measured by Cronbach’s Alpha value is 0.57. According to Hinton and colleagues, an Alpha score above 0.75 is generally taken to indicate a scale of high reliability, and score between 0.50 and 0.75 is generally accepted as indicating a moderately reliability scale (Hinton et al., 2014). The reliability measured by a test-retest design, providing evidence that the KCCPM-55 questionnaire is a valid and reliable tool in the future to determining knowledge about cervical cancer among Arabic speaking population.

Construct analysis of the KCCPM-55 questionnaire illustrated four main domains. In the large field study, one of those had an “excellent” Cronbach alpha value, and three demonstrated moderate reliability of section validity. The “general knowledge about the disease” displayed the least Cronbach Alpha value. In future versions, a partial modification will be considered to improve its internal consistency, maybe by adding more categories in this section. A test-retest analysis of the questionnaire revealed good reliability. However, it is highly recommended that future studies validate questionnaire responsiveness to change over time in responders, this might be tested to predict an increase in women’s knowledge following an educational program.

The main limitation of this study was the sample recruited. The KCCPM-55 questionnaire is tested in only Omani women. Mostly there was a lack of diversity in the sample, therefore, there is a need for evaluation within more diverse populations. It is believed, however, that the results of this study offer a reasonable starting point for planning an educational campaign in Oman aimed at increasing awareness about cervical cancer. The use of this tool in other Arab countries or Arabic speaking population is recommended to obtain a comparison between the findings. Future users can utilize this tool to evaluate the change in knowledge after educational campaigns. However, this purpose must be further tested. 

The KCCPM-55 questionnaire demonstrates the potential to be a useful tool to measure the awareness of cervical cancer, its risk factors, and methods of prevention among women aged 20 years and over. The content of the questionnaire was considered acceptable and valid. The internal consistency and test-retest reliability for the Arabic version was good. Although more research is needed to strengthen the findings, preliminary findings suggest that this tool would be a good instrument to measure the change in knowledge after the educational program.
